# Key Drivers in Reducing Hospital-acquired Pressure Injury at a Quaternary Children’s Hospital

**DOI:** 10.1097/pq9.0000000000000289

**Published:** 2020-04-07

**Authors:** Andrea K. Johnson, Jenna F. Kruger, Sarah Ferrari, Melissa B. Weisse, Marie Hamilton, Ling Loh, Amy M. Chapman, Kristine Taylor, Jessey Bargmann-Losche, Lane F. Donnelly

**Affiliations:** From the * Center for Professional Excellence and Inquiry, Lucile Packard Children’s Hospital Stanford, Palo Alto, Calif.;; † Patient Care Services, Lucile Packard Children’s Hospital Stanford, Palo Alto, Calif.;; ‡ Center for Pediatric and Maternal Value, Lucile Packard Children’s Hospital Stanford, Palo Alto, Calif.; and; § Department of Radiology, Stanford University, School of Medicine, Palo Alto, Calif.

## Abstract

**Introduction:**

Despite being a participating Solutions for Patient Safety (SPS) children’s hospital and having attempted implementation of the SPS hospital-acquired pressure injuries (HAPIs) prevention bundle, our hospital remained at a HAPI rate that was 3 times the mean for SPS participating children’s hospitals. This performance led to the launch of an enterprise-wide HAPI reduction initiative in our organization. The purpose of this article is to describe the improvement initiative, the key drivers, and the resulting decrease in the SPS-reportable HAPI rate.

**Methods:**

We designed a hospital-wide HAPI reduction initiative with actions grouped into 3 key driver areas: standardization, data transparency, and accountability. We paused all individual hospital unit-based HAPI reduction initiatives. We calculated the rate of SPS-reportable HAPIs per 1,000 patient days during both the pre- and postimplementation phases and compared mean rates using a 2-sided *t* test assuming unequal variances.

**Results:**

The mean SPS-reportable HAPI rate for the preimplementation phase was 0.3489, and the postimplementation phase was 0.0609. The difference in rates was statistically significant (*P* < 0.00032). This result equates to an 82.5% reduction in HAPI rate.

**Conclusions:**

Having an institutional pause and retooled initiative to reduce HAPI with key drivers in the areas of standardization, data transparency, and accountability had a statistically significant reduction in our organization’s SPS-reportable HAPI rate.

## INTRODUCTION

It is well known that hospital-acquired pressure injuries (HAPIs) are a significant source of morbidity, pain, patient dissatisfaction, and unnecessary increased cost in the hospital care of children.^1–10^ HAPIs can result in permanent damage to the skin and underlying tissues. A single HAPI has been shown to add approximately $43,180 to hospital care of a child.^3,4^ Other estimates indicate that the costs of HAPI in the United States could exceed $26.8 billion.^4^ Despite being a preventable hospital-acquired condition (HAC), HAPIs occur in approximately 1.4% of hospitalized infants and children.^7–9^ In children, HAPIs include both those related to immobility and medical devices.^1–10^ Some reports show that up to 27% of patients in pediatric intensive care units experience HAPIs.^10^

Following the HAPI prevention bundle (a group of actions which together have been shown to reduce the incidence of HAPI), individual hospitals demonstrated a reduction of serious harm.^1–10^ The Solutions for Patient Safety (SPS) network recommends a 3-pronged approach to the identification and prevention of HAPI: (1) conduct active surveillance to detect pressure injuries, (2) implement and measure compliance with the HAPI prevention bundle, and (3) deployment of a wound ostomy nursing team.^1^ Thirty-three hospitals that participated in the SPS phase 1 initiative to reduce HAPIs saw a statistically significant reduction in SPS-reportable HAPIs (stage 3, 4, and unstageable). These participating hospitals achieved an 80% compliance rate with the established HAPI prevention bundle.^1^

Despite being a participating SPS children’s hospital and having attempted implementation of the SPS HAPI prevention bundle, our hospital remained at a HAPI rate that was more than 3 times the mean for SPS participating children’s hospitals. Specifically, the baseline rate of serious pressure injuries was 0.32 per 1,000 patient days when compared with the SPS benchmark mean rate of 0.09. Quality and nursing leaders paused all current unit-specific HAPI activities due to a lack of consistent bundle compliance and standards. We then launched an enterprise-wide HAPI reduction initiative. The purpose of this article is to describe the improvement initiative, the key drivers, and the resulting decrease in the HAPI rate. The goal of this improvement initiative was to reduce the SPS-reportable pressure injuries by 10% in the first year by implementing an action plan with key drivers determined by a causal analysis.

## METHODS

An improvement initiative to decrease SPS-reportable pressure injuries was organized around a problem-solving template utilizing a key driver diagram with subactivities placed into 3 groupings: standardization, data transparency, and accountability. This improvement initiative was carried out at Lucile Packard Children’s Hospital, Stanford (LPCHS). The system operates approximately 397 licensed beds in a free-standing children’s and maternity hospital, associated ambulatory services, and is a wholly owned subsidiary of Stanford University. Of those 397 beds, 36 are beds managed by LPCHS at other sites, and 49 are related to maternity at LPCHS. There are 312 pediatric beds at LPCHS, and these are the beds used in calculating the HAPI rates. Based on institutional guidelines, this project was considered quality improvement and not human subjects research. Therefore, it did not require institutional review board review and approval.

**Fig. 1.**
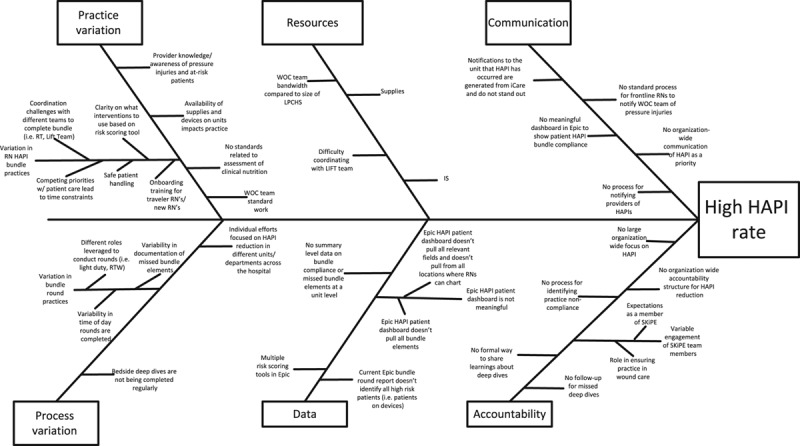
Causal analysis shows contributing factors to a high HAPI rate. RT, respiratory therapist; RN, registered nurse; RTW, Return to Ward (duty); WOC, Wound ostomy and continence nurse; IS, information services.

**Fig. 2.**
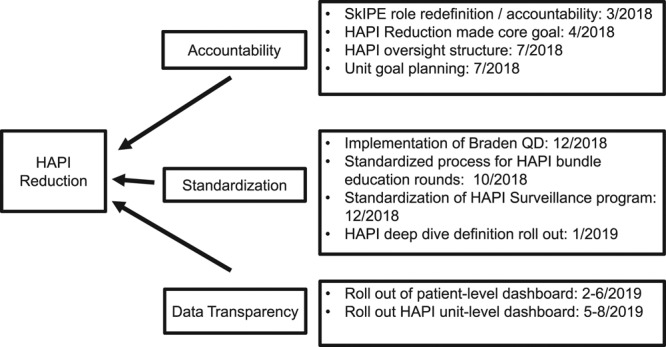
Key driver diagram showing key areas of work in accountability, standardization, and data transparency with contributing actions and dates those actions were activated.

The improvement initiative team performed a causal analysis to determine contributing factors to inconsistent HAPI bundle compliance and monitoring of practice (Fig. 1). We grouped causes into the following categories: practice variation, process variation, data, communication, accountability, and resources. By studying the causes, we created a key driver diagram for the HAPI reduction initiative with activities placed into 3 groupings: standardization, data transparency, and accountability (Fig. 2). This structure mirrors previous organizational efforts that showed a significant reduction in central line-associated bloodstream infections.

Because different subinitiatives were implemented over time, it is difficult to determine an exact date to differentiate the pre- from the postintervention period. For this review, the baseline data for the preimplementation period were from January 2017 through June 2018. The postimplementation period was from July 2018 through August 2019. We implemented interventions on a rolling basis throughout the postimplementation period (Fig. 2). The month of July 2018 was chosen as the pivot date as organizational goals and resources were determined then for the new fiscal year goal supporting HAPI reduction. With the prioritization of HAPI reduction, individual inpatient units developed unit-based HAPI reduction goals in alignment with the organizational initiative. The initiative was led by the leader of our wound care team, which consists of certified wound ostomy nurses in partnership with quality improvement and nursing leadership. The wound care team ensured that the HAPI reduction work was evidence based.

### HAPI Definition and Goal

The purpose of this improvement project was to reduce the rate of SPS-reportable HAPIs per 1,000 patient days. HAPIs included in the SPS-reportable definition are those defined as stage 3, stage 4, or unstageable.^1–10^ The original goal was to reduce the outcome measure, the SPS-reportable pressure injury rate per 1,000 patient days, by 10% in the first year. The definition of reportable pressure injuries used by SPS is consistent with definitions of pressure injuries associated with serious harm used by other organizations such as the National Quality Forum, Centers for Medicare and Medicaid Services, SPS, and many U.S. States.^5,6^

### Standardization—Actions

Within the key driver grouping of standardization, there were numerous subinitiatives to drive standardization of both process and practice. We assembled a group to evaluate the current state of standardized implementation of the SPS HAPI prevention bundle. An interdisciplinary team, including representatives from our Shared Governance Councils, used a combination of evidence-based practice and lean methodology^11^ to reach consensus on standard practice. The representatives included the hospital-wide Skin Integrity Prevention and Education (SkIPE) team which is a unit-based skin team and representatives from Clinical Practice Council, Technology and Informatics Council, as well as certified wound and ostomy nurses, respiratory therapists, and information services and quality improvement staff. This team met and reviewed the best practice literature for HAPI prevention, determined and implemented evidence-based interventions for each of the HAPI prevention bundle elements, and evaluated the feasibility of the bundle interventions in a variety of clinical settings (acute care to critical care). After a review of the literature, the group voted to implement the standard SPS HAPI prevention bundle with the addition of nutrition consults as appropriate in high-risk patients. Defined elements of the HAPI prevention bundle included full head to toe skin assessment, device assessment and rotation, patient positioning, appropriate bed/pressure-redistributing surface, moisture management, and nutrition consult for at-risk patients (Table 1).

**Table 1.**
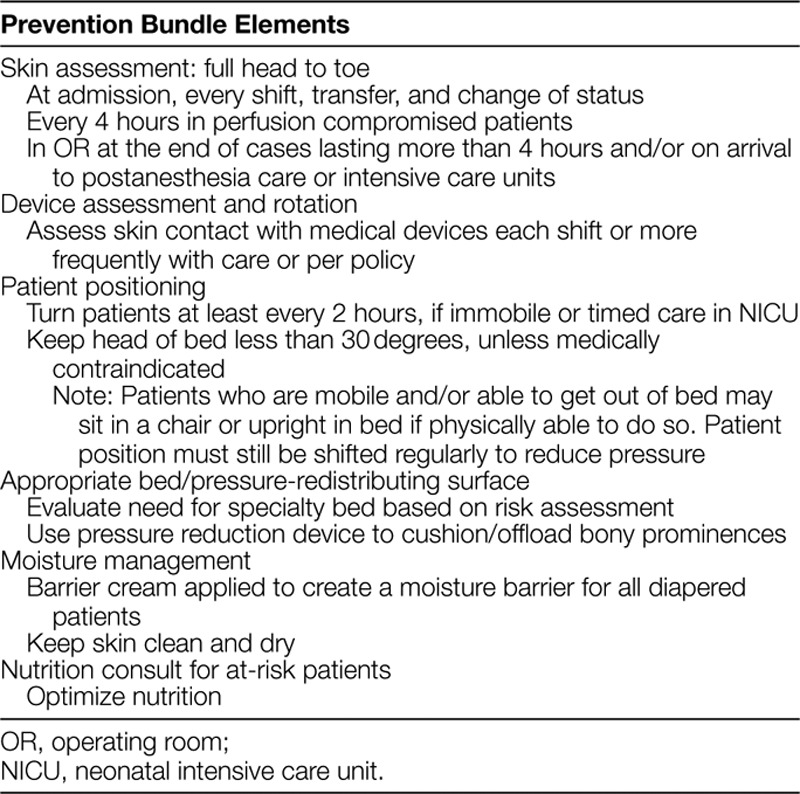
Defined Elements of the HAPI Prevention Bundle Used During Postimplementation Phase

As part of this assessment, the team defined standard processes for HAPI bundle education rounds. Historically, bundle education rounds were conducted variably across inpatient units. The team redefined bundle rounds for HAPI. Specifically, unit-based leadership teams dedicated a minimum of 1 h/d, Monday to Friday, on bundle education rounds. Bundle education rounds focus on compliance with the bundle elements, identification of barriers to practice, and real-time education. These efforts highlight leadership opportunities to remove barriers, create accountability to practice, and demonstrate unit-level support to organizational goals.

At our institution, there has been inconsistent completion of deep dives historically. An interdisciplinary group convened to look at the current state of deep dives for HAPI and to redesign a standard process for all units. Our institution utilizes deep dives after the occurrence of HAPIs (stage 3, 4, unstageable, and deep tissue injury) to identify modifiable and nonmodifiable factors that resulted in the HAPI occurrence. These deep dives require a team approach and involve the unit clinical nurse specialist, bedside nurses, unit management, and physicians, in coordination with the wound ostomy team. The deep dive process is completed partially by the unit and partly by the wound care team. The unit is responsible for following up with those who provided care for the patient and collecting information about factors that may have contributed to the event. The wound care team identifies the source of the injury through patient activities in the days before the event in conjunction with the location and severity of the injury. The wound team also looks at systemic issues or concerns that may have resulted in a higher likelihood of injury. With the newly designed process, for each SPS-reportable HAPI, there is a deep dive investigation to evaluate potential deviations in care that contributed to the event. The deep dive involves review by both certified wound and ostomy nurses, as well as unit-based leaders. The wound care nurses serve as subject matter experts and identify the suspected cause of the injury, potential contributing factors, and deviations in standard care. The deep dive looks more broadly for additional risk factors and practice deviations, such as identifying what interventions were in use, the overall patient condition, Braden QD score,^7^ and time in the operating room. Additionally, the deep dive identifies barriers to prevention efforts and steps that may prevent HAPIs in the future.

Historically, several risk assessment scales were in use within the healthcare system. One of the initial interventions was to standardize the use of a single risk scale for all inpatients, the Braden QD scale.^7^ This action has components of both data transparency and standardization. The Braden QD Scale is an updated risk scale that accounts for risk from both immobility and use of medical devices. It has been validated for use in preterm infants through age 21 years.^7^ Medical devices account for a significant percentage of pressure injuries in children, and the Braden QD allows for quantification of that risk related to medical devices.^7^ To standardize across the hospital and reduce confusion with using multiple scoring tools, we decided to utilize the Braden QD scale for all inpatients, even if over the age of 21 years. The team launched an education campaign for the Braden QD.

There is also the standardization of HAPI surveillance. Active surveillance occurs twice per month in the areas where the highest percentage of HAPIs occur in our organization, the cardiovascular intensive care unit and the pediatric intensive care unit. Nurses who have been trained by the wound ostomy team perform head to toe skin assessments on each patient within the unit in conjunction with bedside nurses. These assessments are opportunities for just-in-time coaching on HAPI reduction as well as identification of any potential or actual pressure injuries. This process is designed to be educational, so the main focus of active surveillance is coaching and modeling best practices while bringing special attention to pressure injury prevention on a routine basis.

### Data Transparency—Actions

Within the key driver grouping of data transparency, there were numerous subinitiatives to drive the availability of real-time data. Initial efforts focused on bringing clarity to data entry and reports within our electronic health record (Epic, Madison, Wis.) to define and record HAPI bundle compliance better.

The organization has a patient-level dashboard in Epic that displays each of the HAPI prevention bundle elements and whether they are compliant/noncompliant in real time. The team revised the elements within the HAPI prevention bundle patient dashboard to match the updated HAPI bundle and interventions. A smaller workgroup comprised staff from information services, wound care (consisting of certified wound ostomy nurses), and bedside nursing met several times to define the specifications for the information services Epic build, focusing on the measurement for each of the bundle elements. After this build was complete, a pilot was conducted with the SkIPE and wound care teams to solicit additional feedback and modifications. The outcome was a unit-level dashboard that aggregates the individual bundle compliance for each patient for each shift on a given unit. This dashboard enables the inpatient units to monitor their HAPI bundle compliance by individual bundle element. It allows units to easily review patients who have had HAPIs for any missed bundle elements. The transparency of data from the individual patient level to the aggregate unit level allowed unit leadership and the wound care team to easily identify trends or gaps in the HAPI bundle elements and address them in real time. The Braden QD Scoring was also incorporated into the HAPI bundle round report in Epic for ease of use in the identification of patients at risk for HAPIs for unit leadership to more effectively target staff for bundle education rounds and allocate resources to the patients most at risk.

### Accountability—Actions

Within the key driver grouping of accountability, there were numerous subinitiatives to drive both accountability and communication. For our fiscal year 2019 (September 2018–August 2019), HAPI reduction was a core quality and safety goal for the organization. This goal was a shift from prior years, where a HAC aggregate metric had been the focus of our organization. This shift in focus from a HAC aggregate to HAPI reduction resulted in significantly more attention and engagement in HAPI reduction efforts. Accordingly, inpatient units developed their own HAPI reduction goals, beginning in July 2018. An email was sent to all staff from the executive offices to commend units for their commitment to HAPI reduction, make staff aware of the planned organization-wide efforts to support HAPI reduction, and request that additional unit-based nonenterprise-wide interventions not be deployed without involvement of the enterprise-wide HAPI reduction team.

We created a HAPI Oversight structure within our quality and safety governance structure. This structure included the creation of the enterprise-wide HAPI reduction team, monthly report outs from that team on the progress in standardization, data transparency, and accountability groups to the hospital HAC Steering Committee. It also included the establishment of a HAC Leadership Meeting in which the leaders of the enterprise-wide HAPI reduction teams reported out monthly on progress and barriers to quality and nursing leadership.By providing visibility to the HAPI reduction activities, the structure helped drive accountability and allow hospital leadership to provide resources or support when the team readily encountered barriers.

Another area of focus for the accountability key driver was the restructuring of our unit-based SkIPE team. The SkIPE team members, a unit-based resource for skin and wound concerns, ensured the use of appropriate prevention efforts, provided education for areas of individual practice deviation, and facilitated the implementation of nursing practice changes related to pressure injury prevention. As part of our improvement initiative, changes were made to improve the accountability of the SkIPE team by creating and enforcing clear expectations in terms of meeting attendance, activities on their units, and participation in pressure injury prevalence surveys. Additionally, the wound care team worked with individual unit managers to reinforce the accountability of SkIPE team member expectations.

### Statistical Analysis

We calculated the rate of SPS-reportable HAPIs per 1,000 patient days during both the pre- and postimplementation phases and compared mean rates using a 2-sided *t* test assuming unequal variances. Statistical significance was defined as a *P* value <0.05. We performed statistical process control analysis with the use of Shewhart control charts. Based on the control limits from the preimplementation period, we looked for patterns in the postintervention period that would permit us to invoke control chart rules that indicate a sustained decrease in the mean of the process. The control limits were modified at the point of implementation, to show process change. The evaluation was performed using Excel (Microsoft, Redmond, Wash.).

## RESULTS

**Fig. 3.**
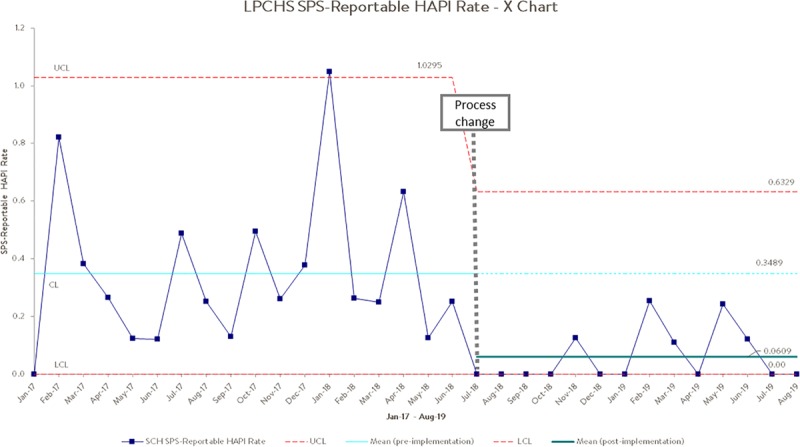
Control chart showing SPS-reportable HAPI rate at LPCHS. The black vertical dotted line represents the time of process change. The turquoise line represents centerline based on preimplementation data. The dark green line represents centerline postimplementation. The dashed red line is the upper confidence limit and lower confidence limit.

The results are shown in Figure 3. The mean SPS-reportable HAPI rate for the preimplementation phase was 0.3489 and for the postimplementation phase was 0.0609. The difference in rates was statistically significant (*P* < 0.00032). This change equates to an 82.5% reduction in HAPI rate. This well exceeded the project goal of a 10% reduction. The number of significant pressure injuries during the preimplementation phase was 49, and the postimplementation phase was 7. This reduced HAPI rate has been sustained thus far through March 2020.

The centerline (mean) displayed on the control chart is calculated based on data from the preimplementation period (mean = 0.3489), and these data determined whether there was statistical process control. The centerline for the postimplementation period (July 2018 to August 2019) is shown in green (mean = 0.0609). In the preimplementation period, 1 point (January 2018) is above the upper confidence limit, signifying special cause variation. In the postimplementation period, all 14 consecutive points fall below the centerline from the preintervention period, satisfying the Shewhart process change rule of at least 8 consecutive points on 1 side of the centerline, signifying a statistically significant shift in the centerline postimplementation.

## DISCUSSION

The focus on HAPI reduction at our organization has been on SPS-reportable injuries. Because of the associated morbidity and cost of these HAPIs, many organizations target the reduction of HAPIs in these stages. These organizations include the National Quality Forum,^5^ the Center for Medicare and Medicaid Services,^6^ SPS,^1^ as well as many U.S. health departments. Despite our initial efforts to adhere to the SPS 3-pronged approach,^1^ we were unsuccessful in reducing our baseline SPS-reportable HAPI rate, and it remained well above the SPS average. Our institutional rate significantly decreased after we paused individual unit HAPI reduction efforts and implemented an organization-wide improvement initiative with key drivers in the areas of standardization, data transparency, and accountability with many action items in each of those areas that. Standardization provided 1 standard screening tool, clear alignment between bundle elements and interventions, and real-time education. Data transparency provided bedside clinicians visibility to HAPI bundle compliance rates and unit leadership a modality to compare bundle compliance with HAPI rate. The accountability provided clear strategic alignment across the organization, governance structure for oversight and implementation, and reset of the SkIPE team membership.

This study has several limitations. First, as the HAPI reduction improvement initiative consisted of many different subinitiative action items which were launched in a staggered overlapping fashion, it is not possible to attribute which action items contributed the most to the HAPI reduction. Such a “bundle” approach is not uncommon with improvement initiatives. Related, the roll-out of the subinitiative actions occurred in a busy pediatric healthcare system with many competing other initiatives and projects. Therefore, because of issues with the timing of allocation of resources, such as information services, it was not possible or ever envisioned that all of the action plans would be enacted simultaneously. The actions were rolled out over time in an overlapping fashion. In this retrospective look back to review the effectiveness of the overall initiative, the point in time separating the preinitiative to the postinitiative time frames is somewhat arbitrary. July 1, 2018, was chosen for the reasons described in the Methods.

Despite these limitations, the overall initiative had a substantial impact on decreasing the HAPI rate. The reduction of the HAPI rate by 82.5% well exceeded the initially established goal of a 10% reduction and was also statistically significant. This reduction shows not only a direct improvement in the care that we provide to children but has also had a positive impact on other processes. The HAPI reduction success was used as an example of an improvement project for our hospital’s recent successful application for Magnet certification and also will have a positive impact on external evaluation processes such as the U.S. News and World Report process for ranking of children’s hospitals. For all of these reasons, the success has been a source of pride for the staff and physicians who work in our healthcare system. We hope that success has also engaged our workforce so that they will be more apt to embrace other improvement initiatives launched in the future openly.

## DISCLOSURE

The authors have no financial interest to declare in relation to the content of this article.
